# "The Hospital" Nursing Mirror

**Published:** 1899-08-19

**Authors:** 


"The Hospital" Nursing Mirror.

Being the Nursing Section of "The Hospital."

[Contributions for this Section of "The Hospital" should be addressed to the Editor, The Hospital, 28 & 29, Southampton Street, Strand, London, W.C., and should have the word "Nursing" plainly written in left-hand top corner of the envelope.]

### Notes on News from the Nursing World.

### THE NURSES' QUARTERS AT THE VENTNOR HOSPITAL FOR CONSUMPTION.

The new block of the National Hospital for Consumption at Ventnor, which was opened by the Princess Henry of Battenberg last week, includes complete accommodation for the requisite staff of nurses, with sitting and bed rooms, kitchen, and offices arranged in the north of the block. Those who on the occasion of the opening ceremony inspected the provision made expressed themselves as thoroughly satisfied with it. The necessity for meeting the needs of the nurses is emphasised by the statement of the chairman of the board of management that there are now 155 beds in the hospital, and that no less than 800 patients can be received during the year. As to the urgent want of the handsome addition to the institution, it is sufficient to say that at the present moment there are 137 persons waiting for admission.

### A CHANGE OF TERMS IN THE TRAINING AT THE HANTS COUNTY HOSPITAL.

In order to allow of the nurses having three full years in hospital, an important change has been made in the terms for training probationers at the Royal Hants County Hospital, Winchester. The old terms were an engagement for three years, two of which were spent in hospital and one on the private nursing staff. The entrance fee was £10 10s.; the salary for the first year was *nil*, for the second £12, and for the third £18, with an extra allowance when private nursing of 2s. 6d. per week. Under the new conditions, now in operation, the engagement is for four years, three of which are spent in hospital. The entrance fee is £5 5s.; the salary for the first year is £6, for the second £12, for the third £16, and for the fourth £25. As before, uniform and laundry are provided, and, if the nurses wish, half of the premium to the Royal National Pension Fund is paid. Although the hospital authorities cannot at present afford to forego some private nursing they have done their best to make their terms as liberal as possible. A more complete plan of lectures and examinations, which will be of great benefit to the nurses, has also been arranged. It may be added that probationers are taken at the age of 22.

### THE NEW NURSES' HOME AT NORWICH.

We are informed that the generous president of the Norfolk and Norwich Hospital, Lord Leicester, has promised to add to his recent gift of £5,000 for a new nurses' home any additional amount that may be required to finish the building. Lord Leicester wishes that the home may be erected as soon as possible.

### A PERNICIOUS PROPOSAL.

A proposal has been adopted by the Gorton District Council to which it may be confidently anticipated the Local Government Board will refuse to give their sanction. The Council have authorised the medical officer "to engage nursing assistance in cases where removal to hospital is considered unnecessary." This sounds very well, but it appears that the kind of assistance contemplated is not that of a trained nurse, but of "some respectable woman who is to be called in to the assistance of the mother of any family in which serious cases of sickness occur." A member of the council urged that the arrangement "would open the door to a lot of quackery," and said he did not think the Local Government Board would approve of it; but as his remonstrances fell on deaf ears, it remains for the supreme authority to intervene. No matter how respectable a woman may be, she is not competent to assist in serious cases of sickness if she is not a trained nurse, and the proposal of the Gorton District Council is as pernicious as it is unprecedented.

### THE MEDICAL SUPERINTENDENT AND THE NURSES AT CAMBERWELL INFIRMARY.

The medical superintendent at Camberwell Infirmary characterises the "press reports about the nurses as 'absurd.'" The matters, however, upon which we commented were statements made by various guardians at their meeting, and we do not see how such statements can have been invented. But, we gladly publish the explanation given by the medical superintendent, which puts a very different aspect on the state of affairs. According to Mr. Keats, "of the eight resignations sent in three were those of head of nurses, who said they had been very comfortable and had no complaints to make. Two of the three had obtained better posts, and one left for 'family reasons.' Of the five others three had obtained better-paid posts; one had gone in for hospital training; and one was a temporary assistant nurse, who was required by the committee to send in a week's resignation, *i.e.*, to go with a week's notice. All said they had been comfortable and had no complaints to make. The nurses reported for 'frivolous complaints' were guilty of neglect in attending to the cleanliness of some 20 out of the 26 patients under their care, whereby the patients suffered greatly—one had an inflamed ear through the unwashed dirt of days' standing." As to the allegation that a nurse was reported because a 'bus conductor lifted his hat to her, Mr. Keats says that "the misinformed guardian had got hold of quite a wrong tale"; and, concerning the alleged plague of rats, his emphatic comment is, "Rats are never seen in the nurses' quarters, and those who perform their duties here conscientiously have nothing to fear but praise and promotion." The phrase is an odd one; but assuming the explanation of the medical superintendent to be correct, there seems to be no reason why good nurses should not apply for appointments at Camberwell Infirmary.

### NOTTS NURSING FEDERATION.

It is stated that a scheme is being considered whereby all the nurses in the county of Notts will participate in the benefits of a pension fund. We reserve comment on the scheme until the details are published, merely observing that many of the nurses at work in Nottinghamshire are probably already members of the Royal National Pension Fund. It is satisfactory, however, to learn that four additional nurses have been lately added to the staff of the federation, and that it is in contemplation to station nurses at Bramcote, Calverton, and Thurgarton. The subject of dress has occupied the attention of the committee, who have been considering the advisability of abandoning the distinctive costume of the senior nurses. Hitherto they have worn pink, and the juniors blue. But it has, apparently, been decided that blue is in future to be worn by all.

### THE TRAINED NURSE IN THE COUNTRY.

It is often in the commonplace work of a nurse's daily life that she earns the greatest gratitude. The following examples of the work of the trained nurse in the country have recently come under our notice. They afford illustrations of the advantages of her presence in isolated districts, and show that it is not only amid the excitement of hospital life that good work may be done. The father of a family, a tradesman, a few Sundays ago got wet in going to church, and unwisely sat in his damp clothing. This imprudence resulted in a sharp attack of rheumatic fever, and his life was seriously endangered. A nurse was telegraphed for, and one was sent, who immediately took charge of the case. The patient's wife, burdened with the cares of the household and shop, rendered what assistance she could. But it was the nurse's knowledge and skill, and the care she exercised in carrying out the doctor's directions, which practically saved a valuable life. The other case was that of a parlourmaid who became suddenly very ill. After a little difficulty a nurse was obtained, who won golden opinions from everyone. The illness lasted several weeks, but eventually the girl became convalescent, and she is now quite well again. Her master and mistress often speak of the comfort and help the nurse afforded them, and of her cleverness and skill in nursing. Without her aid the patient, they say, could hardly have recovered.

### THE BATH BOARD OF GUARDIANS AND THE MIDWIFERY NURSE.

The Local Government Board have been asked by the Bath Board of Guardians to reverse their refusal, and to sanction the continuance of Mrs. Gratte in the office of midwifery nurse "for three months on trial." It seems that during the five weeks she has served Mrs. Gratte has discharged her duties to the satisfaction of the medical officer and the superintendent nurse. But Dr. Ellis, one of the Guardians, and Miss English, with a minority of the Board, are opposed even to the temporary appointment. Whatever may be the qualifications of Mrs. Gratte, Dr. Ellis did well to insist that the question is a serious one, and to point out that "a midwifery nurse may be said to have the life of the patient in her hands." It is extremely unlikely that the Local Government Board would have declined to confirm the appointment in the first instance unless they had good reasons for pursuing that course.

### THE CONGRESS OF THE SANITARY INSTITUTE.

Several subjects of interest to nurses will be discussed at the conference of ladies on Domestic Hygiene, which will be one of the features of the Congress of the Sanitary Institute at Southampton on the 30th and 31st. For example, Miss Rosetta E. Gardiner will raise the question of "The Work of Women Health Visitors"; Miss Smith, an inspector of the London County Council, will read a paper on "Some Causes of Infantile Mortality in England"; and Miss C. J. Wood will introduce the topic of "Crêches and Nurseries for Infants and Children." The conference will be opened by an address from the President, Mrs. Patey.

### A NOTE FROM THE NIGER.

The following matter from a nurse on the spot gives an idea of some of the hardships of nursing in the Niger district: "We are quite out of the bounds of civilisation, about 500 miles up the river Niger. A year ago no white woman had ever been seen here, so we (myself and two other nurses) are objects of curiosity to the natives. We are out in a clearing in the bush—the hospital is one large wooden hut, and our house is another, partitioned off into three rooms. Our patients are officers and non-commissioned officers of the West African Frontier Force, and are all white men, and the cases are mostly malaria and dysentery, for this climate is a deadly one. We took more than six weeks getting here, as the river was so low that we stuck half way, and had to be taken off to a little station. We stayed there over a fortnight, till the river rose enough for us to continue our journey. This is altogether the roughest life. We live almost entirely on tinned foods, but now we are on short commons, as the river has been so low 'they' have not been able to get the stores up. We have no furniture except a camp bed each, and some packing cases for tables. At the termination of the rainy season our men expect to have a good deal of fighting with the natives, so I suppose we shall have heavy work then. A great many of the natives are cannibals, and after a fight they dig up the men's bodies and eat them. I think it probable that I shall soon be here alone, as the other two will be sent to a hospital about 300 miles further up the river."

### NURSING THE LONDON STREET ARAB.

A Nurse writes: "An amusing incident occurs to me of the sharp sayings of the London street arab. The boy I refer to was a lad of 14, and being a rather bad case of periostitis he had been with us some time, and was a decidedly privileged person. When the new dressers came in to begin their duties, he objected to a stranger touching his poor leg, and indignantly protested that ''e wouldn't have that chap touch him.' When I gently suggested that he should mention the dresser as a 'gentleman' and not as a 'chap' the humour of the situation seemed to overcome his grief, and he shouted back to me, 'Well, if you call him a gentleman, I s'pose you calls yerself a laidy.' The general amusement at this sharp speech, and my own inability to convince the boy that such plainly-dressed women could be 'laidies,' made me undesirous of continuing the argument."

### SHORT ITEMS.

The nurses' home at Sutton Coldfield, which forms the local memorial of Her Majesty's Diamond Jubilee, has been opened by the Mayoress. The cost of the building is £1,671, of which all but about £34 has been raised.—A permanent nurse has been engaged for the parish of Shinfield, and she has been provided by the committee with a bicycle.—A promenade concert was given the other day at Severn Side by the Western Silver Band on behalf of the district nursing association. This has become an annual local event, and enthusiastic support is given.

### Gynæcological Nursing.

By G. A. HAWKINS-AMBLER, F.R.C.S., Surgeon to the Samaritan Free Hospital for Women; Assistant Surgeon to the Stanley Hospital, Liverpool.

(*Continued from page* 258.)

### HOW TO TREAT CERTAIN PATIENTS.

THE condition of the patient's bowels will also be noted as to natural constipation, the character of the stools passed, and the effects of various aperients; and you will see whether she be suffering from piles or any obvious affection of the bowels or genitals. Observations that may appear to you of little importance are at times of great significance to the doctor, to whom must be left the duty of estimating their value. You may, in short, find any medical or surgical complication in the special cases you are called upon to nurse. If the woman is menstruating you will notice the quantity and the nature of the discharge, the presence or not of clots in it, the amount of pain with which it is accompanied, the time of its onset, and when it ceases. When the operation is fixed it will be as well to ascertain the expected date of menstruation and mention this to the medical man. Seeing much more of your patient than it is possible for the doctor to see, you must appreciate the responsibility of noting and estimating symptoms that come under your notice for his benefit. The patient may be pregnant, which she may deny strenuously, or she may be suffering from abortion. She may be a malingerer, only pretending, with more or less skill, to be ailing; or a neurotic, and these are most difficult patients to manage, varying from the ordinary hysterical woman, who is such a nuisance to everybody with whom she is brought into contact, with her crazy nerves and imaginary sufferings, to the mildly-nervous and the actually insane. Such cases require a great deal of management, and you must need be the most judicious in the world if you are to have any success with them. There is no necessity to undermine your influence by telling the patient she is hysterical; but a firm, cheery demeanour will be most successful with them, and a steady moral influence can be exerted on them by a nurse of sense and character which will have the best results. Sympathy is the last thing in the world these require. It is what they live on, and is, in many cases, the one thing that lies between them and recovery. They must be braced, their moral nature stimulated, and their characters strengthened and encouraged by the constant tact and intelligence of the nurse. Alcoholic patients are equally troublesome, and, being the most deceptive creatures alive, if you are able to guarantee that any such case is not obtaining stimulants you will be a great deal cleverer than most doctors and nurses with whom I have come in contact. Only those who have experience of these unhappy cases can form the least conception of the dissimulation and cunning they practice in order to obtain alcohol in any form, or realise the tremendous strain they are to the care and temper of their attendants. So, while you are a third hand for the doctor, you may be a useful eye; and mechanical service will find you not without the need of brains.

### MINOR VAGINAL OPERATIONS.

A large number of the gravest operations are now performed through the vagina. And the vagina is the natural home of many kinds of germs. It is extremely difficult to disinfect it, its walls being full of ridges and depressions, as we have seen in describing the operation of douching, and the external genitals, being covered with hair, are very difficult to cleanse. It is now common with many practitioners before even minor operations about the vagina to shave the genitals, which can be done, after careful lathering, with a guarded or safety razor, without damaging the patient. With women of much susceptibility this is best deferred until the anæsthetic has been given. In the meantime, however, the nurse may cut the hair very short, and if the patient have a bath once a day for two or three days before the operation, well soaping the genitals and cleansing them, they can be got into a fairly clean condition. Beside this, it is desirable for several days before operation to give her a vaginal douche of 1 in 1,000 corrosive sublimate lotion or 1 in 40 carbolic acid (say a quart), the vagina being stretched the while, as we described under douching; and a pad wrung out of 1 in 40 carbolic lotion may be placed over the genitals in the intervals between douching. The douche must be administered on the morning of the operation, as well as the night before, and the patient will have had her bowels freely opened by an aperient given the day before, followed by a soap and water enema the night before and on the morning of operation. This must be administered in time to act before the doctor's arrival. She will also be instructed to empty her bladder. In such operations as curetting and plastic operations on the perineum and vagina, this will be sufficient, for the surgeon will scrub the parts with lotion after shaving the patient on the table. For such cases you will have in readiness a basin containing 1 in 1,000 corrosive sublimate lotion, about a gallon of sterilised water (kept hot and covered with a boiled towel) for the douche, and a plentiful supply of hot water, soap, and new nail brushes for the use of the doctor, as well as a clean kitchen apron for him to wear. It will be necessary, too, to have several trays or clean carbolised meat dishes to hold his instruments, and the materials for preparing carbolic lotion to pour over them. If the instruments have been left with you the night before to boil, you will take care that they have boiled from thirty to sixty minutes before the time fixed for the operation. One or two teaspoonfuls of carbonate of soda, according to the number of instruments required, will be a rough but satisfactory quantity to add to the water (about three drachms to the quart of water). It will prevent instruments rusting, and will help to cleanse them. It is a good point for the water to have boiled two or three minutes before the instruments are placed in it, so that the air will have boiled out, and there will be less liability to rusting of those not plated. The doctor will himself place the instruments in the trays or dishes which have been prepared. The nurse must not forget when all is ready to make her own hands surgically clean, but before doing this she will have to help to place the patient in position. That in general use now is the lithotomy position, and this is contrived by the use of Clover's crutch, which consists of a cross-bar with end pieces by which it is strapped to either leg below the knee, and another strap passes over one shoulder and under the opposite arm of the patient, so hoisting up the knees and flexing the thighs well on the abdomen. The patient will have less tendency to roll to one side if the hands are fastened with wristlets. She will wear a pair of warm flannel drawers and stockings, which will not impede the operation in any way; and her chest will be lightly covered as she lies on the edge of the bed or probably on a table. She will have placed under her a blanket and a mackintosh, half of which lies under her, the remainder being draped over the end of the table, so that it falls into a bucket or bowl to receive the water, &c., from the douche. A chair will have been placed for the surgeon at the end of the table, and he will have standing conveniently at his side one or two tables for instruments. You must not forget the anæsthetist. Place a towel for his use, and a basin beside him in case the patient vomit. You will have left in the bed two or three hot-water bottles for use when the patient is returned after operation. If the operation is likely to be protracted, one or two hot-water bottles placed beside her will help greatly to maintain her body heat. In severe operations it is necessary also to have ready for use a nutritive enema, with some brandy that can be added to it. Some surgeons use for the douche salt water of a strength of one teaspoonful to a pint.

### First Aid Lectures in the Apennines.

By a Nurse.

Eighteen months ago, when I was in England, I was much struck by the potential usefulness of a printed set of rules for "first aid" arranged by Mrs. Clare Goslett. In a few brief words she told what to do until the doctor came in most emergencies. The author kindly gave me permission to translate and spread this card in Italy, so with the aid of an Italian doctor and surgeon a careful rendering was produced and 500 copies were printed. In various parts of Italy there happens to be a movement on foot to enlighten the public on health questions; these little cards consequently have already gained a certain amount of favour. So far, however, their most kindly reception has been in the tiny mountain hamlets known collectively as Abetone, where a friend has arranged with the doctor of the commune (who resides eight miles away) to hold little classes, with peasants as pupils, explaining to them the instructions on the cards, and afterwards putting them through an examination on the same. The first lesson was in many ways amusing, as the doctor wisely sought to promulgate new ideas by explaining the errors of the old, and consequently began by asking his pupils what they would do in the case of such or such misfortune.

Beginning with fainting, he elicited the answer that three out of four would put the patient with the head high, whilst no one would hazard a suggestion as to the position in which an apoplectic person should be placed.

My friend here interposed, inquiring, "What colour is the apoplectic person, and what the fainting one?"

Receiving the answer, "Red or purple the former; pallid the latter," she explained. "Then you will remember that the person with a red face has too much blood to his head, and so must have it placed fairly high that the blood may run down from it; whilst the pale person has too little blood in his head, and so you must lower it that the blood may run up into it."

This explanation, rapid and graphic, impressed itself most distinctly on the dullest brain. At the following examination no one mistook again the position in which they would have to place the two patients respectively.

The doctor next dwelt largely on the duty of getting rid of useless persons in cases of emergency; but there I fear our instructions will bear limited fruit. It is not likely that a poor peasant will have sufficient authority to eliminate the neighbours and relations who crowd around anyone doing something out of the ordinary. However, the pupils expressed their full comprehension of the need of quiet and air, and one can but hope that to some extent they may be able to insure them.

I was much struck here by one of the pupils, maid to my friend, and a woman of remarkable intelligence, asking the doctor, "Excuse me, but is there danger in keeping the window open near a person in an apoplectic fit; as you say we are to loosen his clothing is he not likely to catch a chill?"

This showed entire grasp of the situation. She mentally saw the patient in bed, in a small room, superfluous friends sent away, hours to wait for the doctor, snow very probably outside, and wind blowing on the exposed throat and chest. One could only answer, "avoid actual draught, put hot-water bottles, bricks, blankets to the feet and legs of patient, but ventilation, pure air, is a necessity." In the following examination all this was repeated in a way that showed entire comprehension.

When we came to cuts and wounds the doctor's question, "What would you do?" elicited very strange answers. One declared she had been told to seek for spider or cobwebs and lay them on the injuries; another used potatoes; and a third had seen manure applied. With a shudder we hastened to explain the danger of poison getting into the blood by such practices, urging the peasants in future to boil water for twenty minutes (we fear putting disinfectants in their hands lest children should poison themselves), dip clean bits of linen in the same, with which to wash thoroughly any wound, and afterwards dress it, thus keeping it safe from mischief until the doctor came.

To impress them with the need of knowing what to do in such emergencies we dilated on the sad consequences of leaving a wound in contact with dirty handkerchiefs or rags for possibly many hours. Even though they telegraph at once for the doctor, he may not be at home for hours, having a large district to traverse. The telegraph office, too, closes at seven p.m., so after that hour they must send a cart to fetch the physician themselves, which also means delay. Constantly in winter the roads are blocked with snow, and a whole day and night may pass before he can reach them. Therefore, the advantage of knowing how to apply a tourniquet so as to prevent exhaustive hæmorrhage, and how to wash and dress aseptically the wound so as to prevent poisoning, was easy to demonstrate to them.

"You may be able to prevent, not so much death, as unnecessary length of illness; may help your relations and friends to a speedy convalescence and return to strength, instead of leaving them to endure long inflammation from wounds, and consequent debility, inability to work, and, therefore, insufficiency of food." Here, too, they expressed complete comprehension, and determination to boil water and treat all future wounds with it.

Another point which brought forth matter of interest was that which dealt with "bites of mad dogs or serpents." It seems that there are poisonous vipers in some of the woods here, though no one present remembered any human being having suffered from them. The peril had, however, been brought home to them some years ago by a dog being bitten at Boscolungo, and his life having been undoubtedly saved by the same maid who desired information about the risk of pneumonia for apoplectics. My friend related how she had first known her Fortunata from seeing her marching up and down the high road leading a big dog with a bleeding jaw. This woman had had the courage and nerve to cut away the tissues surrounding the bite (lower jaw), encouraging bleeding (conceive the dog allowing it!), and afterwards, someone having said that it was necessary to keep poisoned persons in motion, she walked the poor beast up and down for twenty-four hours! She saved him, and they both became the happy property of my friend.

I could but bow my head in homage to such superlative nurse qualities. We, with all our instruction and practice, might have failed in courage to carry out such surgical, or the resolution to continue such exhausting medical, treatment. But this simple peasant woman had failed in neither! Who can say after this that it is useless to attempt to teach uneducated people hygiene and "first aid"?

Rare such women as Fortunata necessarily are in every nation, in every class; but since we have fallen on one here, it is surely encouragement to continue the propaganda, in the hopes that here and there, at any rate, our words may fall on hearts and intelligences like unto hers.

### A CORRECTION.

Miss Mary Thomas desires to state that she has been appointed Assistant Matron of the Grove Hospital, and not of the Fountain Hospital.

### Across the Seas.

### NURSING IN FIJI.

(By Our Own Correspondent.)

Fiji is one of the smallest of the British possessions. Its hospital contains one hundred beds, and it is constructed of wood, in separate blocks, on an elevation about a mile from the seat of Government, and overlooks a beautiful harbour, which is bounded on the northern and western sides by ranges of thickly-wooded hills gently sloping towards the bay. The tropical sunsets are seen in their magnificence from the ward verandahs. The many hillsides are still in their primæval beauty, tree ferns and palms grow luxuriantly, and vegetation is green all the year round.

The Europeans' block contains twelve beds distributed in three wards, and here also are the operating theatre, ophthalmic room, office, dispensary, bathrooms, &c. The other five blocks are devoted to native patients, consisting of a medley of Indians, Fijians, Melanesians, and others. The Europeans are, of course, treated as they would be in England, but we have to adopt native customs for the others in so far as is consistent with good sanitation and discipline. The Fijians use the native mats and wooden or bamboo pillows, and when placed on a canvas stretcher or asylum pattern iron bedstead, these articles really look very pretty and quaint, and are particularly striking to strangers. All the floors are waxed, and each ward is surrounded by a spacious verandah, which is much appreciated by convalescent patients. The water supply is unrivalled, and every block is provided with a shower bath, so that many patients who come from districts where this commodity is deficient in quality and amount deem it a great luxury. Our own home is situated about the centre of the site, being near to the medical superintendent's house and the high road, and close to the Europeans' block.

Young Fijian students are trained here, and after a three years' curriculum they are presented for examination, and are eventually sent to tend the sick and engage in sanitary work in the country districts among their own people. During the time they are in the hospital they act as dressers, and soon become skilful in dressing ulcers and wounds, and very readily acquire the art of bandaging neatly.

The heavy ward work is done by Indian and Fijian servants, who also help as attendants in the native wards. The heat in summer-time is very oppressive, and often very exhausting, especially after a long residence in the tropics. We often have almost incessant rain for weeks, and at such times our work is done under many difficulties, the wards being so widely detached. Still, on the whole our lives are very happy and the work is interesting. Apart from pure nursing, the various characteristics of the native races are well worthy of study, and often present some amusing features.

One great difficulty which we have to surmount lies in having to acquire several languages. So many are spoken that it is necessary to have a fair knowledge of them in order to have the work done in a satisfactory manner; Fijian and Hindustani, at least, are almost a *sine qua non*. The dispenser is a native Fijian, who entered as a student several years ago, and he is a most reliable and trustworthy man.

As for our work here, and what we learn by it, I may say that the cases are, as might be expected, mostly of the tropical type, both among Europeans and native races. We sometimes think that there must be scope for a special course of instruction in the nursing as well as in the medical treatment of tropical diseases at some of the larger medical schools at home, where, I am told, the subject is engaging the attention of the nursing staffs and of the Colonial Office, as it affects the training of medical men who expect to go abroad.

We have a good many patients with dysentery. These, thanks chiefly to ipecacuanha, we generally manage to save; and we have not been altogether unfortunate with liver abscesses, of which we get two or three every year. Typhoid is quite rare in Fiji, but cases of the tropical type of it are now and again brought to our hospital from Samoa, where it is more common, and from H.M. ships on the station. Some of our pet patients are, indeed, Bluejackets; and we still have at the present time some of those wounded by Samoan skirmishers at the shelling of Apia in January last.

We are hardly sorry that Fiji is free from malarial diseases, and that there is neither cholera nor plague among us as yet. Our chief speciality is frambœsia, of which, perhaps, English nurses will ask, "What is that?" The answer is "Yaws," which you may look up in the text-books if you like, and find very little there about it. We have always ten or a dozen cases of leprosy on hand, who are quartered in a separate camp of wooden huts in the hospital grounds. About 25 per cent. of our native Fijian patients have filariæ in their blood, the examination of which forms a fascinating study to nurses who have a microscope available, and the time to use it in. Among parasites, the anchylostoma is common in Indian coolies, who are frequently brought in suffering from a severe form of anæmia which the medical officers attribute to the ravages of this little worm. A good many ringworm cases of the kind called here "tokelan," come to us, and are treated with chrysophamic acid or iodine, both of which stain annoyingly, and are apt to worry a tidy nurse to distraction.

We do not get many serious accidents, but we have a fair number of operations in the course of each year (165 in 1898), and existing as we all practically do in the open air, we are free from the septic disagreeables and risks which in a cold climate are apt to assert themselves in the wards of large hospitals in crowded cities. Our death-rate last year was in fact only 3·38 per cent. of all the admissions—40 out of 1,178; and only one of these occurred in connection with an operation, a laparotomy.

Our uniform is, of course, airy, and adapted to the climate; but our friends tell us that they like its simplicity and neatness, and we find it very comfortable. The ward dress is of pale blue-grey zephyr, and the cloak and bonnet are navy blue, of the "New Welbeck" pattern.

### The Unseen Cord.

There is an unseen cord which binds
The whole wide world together;
Through every human life it winds—
This one mysterious tether.
It links all races and all lands
Throughout their span allotted,
And death alone unties the strands,
Which God Himself has knotted.

However humble be your lot,
Howe'er your hands are fettered,
You cannot think a noble thought,
But all the world is bettered.
With every impulse, deed, or word
Wherein love blends with duty,
A message speeds along the cord
That gives the earth more beauty.

Your unkind thought, your selfish deed,
Is felt in furthest places;
There are no solitudes, where greed
And wrong can hide their faces;
There are no separate lives; the chain,
Too subtle for our seeing,
Unites us all upon the plain
Of universal being.

Ella Wheeler Wilcox.

### Hospital Johnnie.

JOHNNIE WHITE could never remember having been anywhere but in the children's ward. He was brought there at three years old with hip disease—a puny thing that looked more like a two-year old sickly baby than a boy of three.

He was a foundling, fondly cared for by an old Irishwoman who called herself his grandmother, and had had a hard fight to obtain a meagre living for herself and the child. Even so she found it a sore trial to part from this child of her adoption when the doctor procured a bed for him in the Children's Hospital. Nurse Edith saw him first when he was six years old, lying on his back white and quiet in the little cot that had been his home for three years.

Edith Mansell had only entered on the new life of a probationer the day before, and was full of love and sympathy for the helpless little ones who surrounded her on all sides. She had come fully realising the important step she was taking, and not shrinking from the many hard and disagreeable duties she would have to perform. Little Johnnie went to her heart at once, and, bending over him, she succeeded by loving, bright words in calling up something like a smile to his prematurely old and sad little face. She felt quite proud of this a few minutes later, when another nurse remarked, speaking of the little sufferer, "Ah, poor Johnnie; he never smiles."

Her duties were light that first day—till evening came—and she was told off to wash all those on one side of the ward; and, then, how her back ached, stooping over the small people, making their beds, &c. She was tall, so felt i more than most. Still she enjoyed the work, and was surprised to find how wonderfully full of life and spirit the children were when not in actual pain. One great topic of conversation between them was the respective doctor who attended them, each stoutly declaring that his or her doctor was the best, and shouting the same across the ward. "Shure Dr. Brown's the best doctor in Cork," said one curly-headed youngster with a broken leg. "Dade thin, Tim Murphy, 'tis Dr. Foster's the best wan intirely; don't I know it meself," shouted a thin, weazened-faced boy, who was at that moment in Nurse Edith's arms while his bed was being made. "Ah, now, nurse darlint," said a third, "tell us, which is the best of thim doctors?" The nurse thus appealed to—evidently a favourite in the ward—said dispassionately that they were all so good, she could not tell which was best—so the discussion ended temporarily, to be renewed at the first opportunity. It need hardly be said, the above took place in an Irish hospital.

Presently a low wail fell on Nurse Edith's quick ear, and looking round, she found it came from Johnnie's cot at the top of the ward, where he was being washed in his turn. The wail became louder and louder, and Nurse Edith could hear him being told he was a "naughty boy." How she longed to go and tend him herself, for she had a feeling that he would not cry with her, though the other nurses said he always cried when he was touched. It was her first experience of the acute human suffering of the world, and it quickened her sympathies and longing to help, and brought the tears to her eyes. "Shure that new nurse knows a dale," said a pale little lad to his neighbour, after being comfortably tucked up for the night. "She bates the rist of thim holler. The way she did hould me—sorra a bit did she hurt."

Next day, when off duty, Edith went out, and came back with a lovely spray of wild roses, which she fastened in her dress; and when leaning over Johnnie she saw his eyes brighten as he fixed his gaze on the flowers.

"Would you like them for yourself, little man?" And so saying, she put them into the tiny white hands, after first removing the thorns, and his pale little face positively glowed with pleasure as he smiled up at her.

"We must be great friends, John. Shall I bring you a flower every day?"

An emphatic nod answered her—he had not found his tongue yet.

"Did you ever have a flower of your own before?" An unmistakeable shake of the head gave the sorrowful reply. "Well, now," she went on, "if I wash you to-night, Johnnie, will you be quite good and not cry? I'll sing to you," she added, "if you are very good." A scarcely audible little "Yes" and a nod rewarded her efforts, and she went to her work with a light heart, feeling that she had already been of use to "one of the little ones."

When bedtime came she sought and obtained leave to wash Johnnie. No one seemed very envious of her, but it was a very bright face the wee boy saw as she brought the necessary things for the ablution. He was just screwing up his face to cry as usual on being touched, but with a "Remember, John," the began to sing in a soft, low voice the children's evening hymn, "Now the Day is Over," and the novelty and the music together had such a soothing effect that not a cry escaped him the whole time. She sang hymn after hymn till she had him settled up cosily for the night, and had put out the poor little leg quite straight, and gently let down the weight attached. No one noticed them or heard her sing; the noise and bustle of the ward at that time prevented such gentle sounds reaching beyond the next cot. Then she bent and kissed the little white brow, and with his precious roses clasped on his bosom, little John went to sleep more quietly than he had done for many a night.

I think it was as good a day's work as a probationer ever did on her first day in hospital—what think you? Do we all give that "cup of cold water," which it is so easy to give, as often as we might? One word more about Nurse Edith and her little patient. After a happy two years at the hospital she left and went to England to carry on her work of love elsewhere, never thinking she should see her little friend again, for he was supposed to be dying when she left. But on visiting the scene of her labours more than a year after, she heard that Johnnie had become so much better—he was going to leave the hospital, and was actually able to walk a little. She arrived the day before he left, and no sooner did she reach the door of the children's ward—although she was not in uniform, and Johnnie had never seen her otherwise—yet he had heard and recognised her voice, and shouted out "Nurse Edith," and when she came to his cot there he was half-hidden under the bedclothes in a fit of shyness. But such a bright little face presently emerged, very different to the one she had left. Would that all the little sufferers recovered as happily as Johnnie White.

### Minor Appointments.

STAFFORDSHIRE COUNTY ASYLUMS INFIRMARY.—Miss Rachel Jones has been appointed Staff Sister. She was trained at the Poplar and Stepney Sick Asylum, Bromley, and has since been nurse at the Northern Hospital, Winchmore Hill, London.

NATIONAL HOSPITAL FOR EPILEPSY AND PARALYSIS, QUEEN SQUARE.—Miss E. Godwin has been appointed Sister. She was trained at the Royal Hants County Hospital, and has since obtained her L.O.S. certificate.

ROYAL ISLE OF WIGHT INFIRMARY, RYDE.—Miss A. Carr has been appointed Charge Nurse of the new Children's Ward. She was trained and has since worked on the staff of the Royal Hants County Hospital.

GUEST HOSPITAL, DUDLEY.—Miss D. Creed has been appointed Charge Nurse. She was trained at the Royal Hants County Hospital, Winchester.

ROYAL HANTS COUNTY HOSPITAL.—Miss E. Hancock has been appointed Ward Sister. She was trained at the same Hospital.

UNIVERSITY COLLEGE HOSPITAL.—Miss E. Walker has been appointed Staff Nurse. She was trained at the Victoria Hospital, Chelsea, and at the Royal Hants County Hospital.

### A Nurse's Life in a Workhouse Hospital.

By ONE OF THE NURSES.

THE workhouse hospital of ——— is a large one, with accommodation for over 800 patients. There is, of course, a matron, and a staff of 48 nurses. There are three doctors, one, the head doctor, living away from the hospital, the other two (one of whom is a lady) being resident.

The term of nurses' training is three years, at the end of which time a certificate is given them if the examination they have to undergo is satisfactory. Their salary the first year is £12, the second £16, and the third £20, with a set of indoor uniform each year. There is one great advantage for a nurse being in this workhouse hospital, she has no menial work to do (unless bathing dirty admissions may be called so), but plunges at once into actual sick nursing without being kept six months or a year at probationary work as in many hospitals. This enables her, if she "has her eyes open" and "reads up," to begin right off to learn a great deal. The work varies as regards heaviness and lightness; sometimes the nurses are almost run off their feet, and at others they have just enough to keep them going comfortably. Two or three serious admissions, or two or three acute cases, dying or becoming convalescent, make a wonderful change both ways.

The nurses live in the home, a building quite distinct, but bordering on the hospital, which is very comfortable and commodious, with lawn and flower beds, tennis court, and seats beneath "the golden laburnum trees." Most of the nurses have a comfortably furnished bedroom to themselves, which they ornament with pictures, bric-a-brac, &c., and where many a time (but "tell it not in Gath," for it's against the rules) half a dozen of them meet for "afternoon tea," sitting some of them on the bed and others on the floor, for there are only two chairs. There is a general sitting-room (with piano) and dining-room. The food is good and the rules are not strict. The nurses can have their friends visiting them in their off duty time, giving them afternoon tea in the sitting-room. Often some of them invite several of the latter, and ask their fellow nurses to meet them. These little jollifications are very much enjoyed on both sides.

The day nurses' "getting up" bell rings at 5.45 a.m., breakfast is at 6.30 a.m., and they are in their wards at 7 a.m. Their time "on duty" is from then till 8.30 p.m., with half an hour for lunch, forty minutes for dinner, and two hours off (one day from 2.30 till 4.30, and the other from 4.30 till 6.30), for which time they have a "pass." The latter gives them egress through the hospital gates. Supper is at 8.30 p.m., the nurses have to be in their rooms at 10 p.m., and put their lights out at 10.30 p.m. Besides the two hours off each day they have half a day a week (from 2 p.m. till 10 p.m.) and a whole day a month (from 8 a.m. till 10 p.m.), when they do not go on duty at all, and moreover are privileged to have their breakfast in bed. The night nurses' "getting up" bell rings at 4 p.m., and tea is at 4.30 p.m. It is optional whether or not they get up at 4 o'clock. Some prefer being lazy and to have tea later on with a chum in their own rooms, which they provide for themselves, boiling their little kettles on the gas. Their passes are from 4.30 to 7.45 p.m. Dinner is at 8 p.m., and the nurses are in their wards at 8.30 p.m. till 8 a.m. the next morning. They have two meals on duty in one of the ward kitchens. Over the night nurses is a superintendent, who pays sundry visits to the wards to see that the former are not asleep and that they are doing their duty. In cases of emergency she is telephoned for, and gives instructions. She also communicates with the doctor if necessary. Breakfast is at 8.30 a.m., and after, if the nurses are wise, they get to bed. Both night and day nurses have half a day a week and a whole day and night a month. The term of night duty is about three months (three terms have to be served in the three years, sometimes four), and it is so trying to most nurses that they are very glad when they are due off.

Though some of the patients are the poorest of the poor and the lowest of the low, the best as regards diet, medical and nursing skill is at their disposal, and many say, "If I'd known it was like this I'd have come long before." A nurse on inquiry found that there was in her ward a young man with a University education, and able to converse with her on Carlyle, Ruskin, &c. Another was a clergyman's son, who for years had been harlequin at the pantomime; a third was leading tenor singer at the cathedral in the city; a fourth was a gentlemanly, intelligent man, a commercial traveller; and a fifth used to occupy a good position, earning £6 a a week. In other wards there were a doctor, a lawyer, a schoolmaster, and a clergyman. Most of these had come down through "the drink." In the women's wards the same sad examples are found. Cases of pathetic interest come to light. One example: An old woman, 79 years of age, by dint of hard saving, had scraped together £50, which she gave to her son, and went to live with him. He appropriated the money and sent his mother to the workhouse. Some of the patients in contrast are very amusing. A widow was dying. The nurse, wishing to be sympathetic and to say something comforting, said, "Oh, well, by-and-bye you will be joining your husband." "Oh, nurse," she replied, in consternation, "I hope not!" She had evidently had enough of him in this world, and did not want any more of him in the next. The midwifery ward reveals many a tale of shame and sorrow, for the bulk of the patients are girls who have "got into trouble." Many of them are of the servant girl type, but some are of the better class. Doctors, lady guardians, nurses, and "visiting ladies" take an interest in these girls, and try to help them to redeem their character. Some are disappointing, but others turn out well.

The wards are airy, light, and bright with pictures, plants, and flowers (the latter provided by the ladies of the "Flower Mission"). The children are so content with toys, dolls' houses, rocking-horses, &c., that they cry when they get their discharge; and no wonder, when some of their homes are in the back slums of the city with drunken parents.

The nurses' training is good, the doctors and matron give lectures, and by the obstetrical lectures and midwifery "cases" the nurses are prepared for the examination of the London Obstetrical Society.

In a nurse's life there are many difficulties and disagreeables to contend with (such as going to the mortuary with the dead in "the drear hour of night," bathing a dirty admission who is "walking away" with "live stock,' dressing loathsome ulcers, cancer cases, &c., &c.); but if she undertakes her work, realising her "high vocation," she will find in it unlimited interest and a source of happiness in the display of tenderness and sympathy towards the poor suffering ones who are placed under her care.

### National Health Society.

THIS society will next month recommence its special course of training lectures for ladies who wish to obtain the certificate of the Sanitary Inspectors' Examination Board recently established under the authority of the Local Government Board, or to qualify as teachers of the laws of health and sanitary subjects under the County Council scheme of technical education, as factory and sanitary inspectors, or in preparation for hospital training.

### Echoes from the Outside World.

AN OPEN LETTER TO A HOSPITAL NURSE.

It was quite nice to get away from London. Everyone except ourselves seemed to have moved off, and we began to feel quite Selkirk-ish in our solitude, and would have been distinctly glad if there had been someone "our right" "to dispute." The nearest approach we found in the tradespeople, who, as soon as August had turned, looked daily more surprised that we had not departed, and appeared almost disposed to dispute our claim to remain in our own house. Now I am writing from a real country retreat, where I am staying for a few days before I join the rest of our party at the seaside. After the long streets of uniform houses of which a big city is so largely composed, the delight of looking out on green field, and gold field, waving wood, and flower-decked common, with only a few cottages here and there peeping up to break the monotony of the scene, impresses me as the best thing on earth, for the time at least. It is not until one comes away from town that one fully realises how tired the eye becomes of being unable to get beyond the next brick wall. I expect that in some way this sensation of fatigue reaches the brain, and that is why, after a long spell of life in London, we fall into such a state of collapse. My host and hostess have, I find, been arranging no end of lovely excursions for me, but my pathetic appeals to be allowed to sit still in the garden and just do nothing but let my eyes and my brain get expanded again after their recent cramped condition have been successful, and I have joined the great army of Do-Nothings which has so many recruits just now, and for the time being am one of the laziest souls on earth.

---

The development of the Dreyfus case threatens to supply holiday-makers with a romance of the most exciting character without the obligation to have recourse to the lending library. The arrival of the daily newspaper, which always seems to be welcomed with such delight, especially by the male element, when they are away from town, will be watched for even more eagerly than usual. At present the prisoner—for such I suppose he remains till he is formally acquitted—appears to be slowly breaking down the barriers of prejudice against him which still exist in many quarters. Numbers who believed Captain Dreyfus guilty have been sorely shaken in their ideas, and now that he apparently begins to trust more in himself and no longer keeps himself under such strong control as he did at first, the improvement in his demeanour is said to be very marked. The dazed look which he wore the first day of the trial is described as being replaced by the alertness more natural to a man trying to seize on every detail which might help to vindicate his innocence, and, of course, the fact of the attempted assassination of M. Labori and the theft of his papers has told enormously on the side of the defendant. It lends such a strong probability to the story of a conspiracy in which the actors, it would seem, have not even yet come to an end of their villainous endeavours to blast his career.

---

Except to a few women of a very objectionable type the news that "Savage South Africa" was going to return at once *en bloc* to the land from whence it came would cause nothing but rejoicing. Most of you will remember that when first this particular feature of the Earl's Court Exhibition was mooted Sir Alfred Milner and other wise men who had the welfare of South Africa at heart did their utmost to discourage the enterprise. But opposition proved useless, and the very disaster which was prophesied has come to pass. White women, whom the Matabeles have been taught to respect and to treat with exceeding deference in their own land, have here proved themselves far removed from the pure-minded, self-respecting individuals the savages have been taught to consider them, and the familiarity with which some of our English women have treated the Matabeles, simply because they are handsome, well-made men, ending in the deplorable incident between Lobengula and Miss Jewell, must have an evil effect upon the future relations of the English and the natives in South Africa. Those who understand the language aver that the remarks made by the black man about their female visitors would prevent any Englishman allowing his wife, sister, or daughter to go near them, had he only the power of ready translation. But because he has not, and because in many cases he is profoundly ignorant of the difficulties of life in South Africa, a state of affairs is allowed to prevail which may ultimately, especially in case of war, have most unpleasant results. The "show" should never have been permitted.

---

I am always much interested in the "Zoo" and its occupants, and two of the recent arrivals are more than unusually worth noticing. Not very long since some one opening a banana discovered a locust, and in a fit of generosity preserved its life, and sent it to the Zoological Gardens. There it was placed in the insect house, and looked upon as a valuable addition to the collection. But now the satisfaction felt seems likely to change into alarm, for already the one locust has multiplied itself into three hundred locusts, and there seems no prospect at present of the eggs ceasing to increase. Those who have had any experience abroad of the awful consequences resulting from the depredations of locusts maintain that it will require a great deal of care to prevent a few stray specimens from making their escape, and should they do so the verdure of the gardens and of Regent's Park would speedily become a thing of the past. But I hope this is only a scare of the silly season, and that a wholesale slaughter will, if necessary, remove any chance of such a disaster occurring in London. The other arrival at the gardens is the pair of grey zebras which King Menelik of Abyssinia has sent over to Her Majesty the Queen. I do not know whether the Abyssinian monarch pays for their transit or whether Queen Victoria has to do so; but, as the assistant superintendent of the Zoological Gardens had to go all the way to Somaliland to fetch the gift, the safe carriage of these beautiful animals must have cost a good deal. I do not fancy that the Queen is likely to depose her dear old donkey in favour of any zebra, however rare, so that I fear King Menelik's desire to forward to the Empress of India a couple of handsome steeds for her own use will hardly be realised.

---

The account which appeared in these pages last week of the delights of an Alpine expedition must have made many of us wish that we too could wend our way to those rocky heights and enjoy the pleasures of "fresh scenes and pastures new." But the long list already existing of accidents on the mountains seems to be receiving many sad additions this year. The death of Miss Bridge must strongly accentuate the feeling that it is impossible to be too careful even where no danger appears to exist. Walking on a grassy slope seems a comparatively simple and safe action—to an inexperienced mountaineer — but the guides who know where danger lurks, bid climbers beware of these inoffensive slopes with their very short slippery grass, and their yawning precipices such a short way off. Miss Bridge would probably have mounted safely had not an insect—most likely a horsefly, so abundant in Switzerland and Northern Italy—stung her so severely that she started, lost her balance and met her death. Those who know the Alpine district say that it was an accident which might have happened anywhere, but Zinal has been particularly fruitful in accidents to ladies, though many of the deaths have been occasioned by falling stones rather than by fatal slips.

### Typical Patients.

### THE INFIDEL PATIENT.

Maria Flipkins was admitted to the A Ward of the L—— Hospital suffering from erysipelas of the face. She had not been half a day in the ward before most of the patients had heard her family history and list of troubles, and had agreed with her that she was the most unfortunate person that had ever been born.

She had a piece of strapping on her nose that gave her a weak, vacillating expression which did not inspire confidence. She was always talking of that dreadful fall she had had, which had injured her nose. "The wonder to me is that it wasn't broke, an' all my friends said the same," was the usual termination to the "nose" story.

The lady who came twice a week and delivered a religious discourse to the patients found Maria Flipkins a sad trial to the flesh. She refused to sing hymns. She said her husband had always been "down on 'ims." Why, had she not been singing "Brightly Gleams our Banner" when they brought "John Thomas 'ome a corpse!"

"Yes," exclaimed Maria, with supreme self-importance, "I've never sung an 'im since; an' as for settin' foot in a church—you might put me through a mangle first."

Maria's temper, which was not of the sweetest at any time, was very much aggravated by her painful complaint. "I'd like to know 'ow you'd feel with your face swole up to the size of a pumpking," she grumbled, when Miss Cole (the religious lady) came over to speak to her after the service.

Miss Cole coughed and looked sympathetic. "These trials and afflictions——" she began, in her plaintive voice.

"Rats!" muttered Maria, under her breath. "I don't b'lieve in religing at all, not since all them things 'appened at 'ome. I've bin a hinfidel ever since."

"What were the troubles?" inquired Miss Cole, feeling extremely sympathetic.

"Oh! Tommy scalded 'isself on the Toosday, an' Ellen Mary got the netling rash; and there was poor John Thomas dead an' coffined afore Sunday; an' Uncle Peter broke a blood vessel in the back kitchen on the Monday, so 'e an' John Thomas was both buried on the same day. And then I fell down an' might 'ave broke my nose—an' all my friends said it was just a chance that I didn't—an' now 'ere comes this herysipelas to torment me to death."

"Are you prepared to die?" asked Miss Cole, getting more plaintive in her anxiety.

"Didn't I tell you I was an hinfidel," snapped Maria. "Uncle Peter was the only Christian in our family. He said it was very 'ard work—I was reel set on Uncle Peter—I never took so much trouble over anyone's collars as I did over 'is. I often ses to 'im, 'Peter,' I ses, 'you won't get your collars starched so well when you git to the other place.'"

"This is blasphemous," exclaimed Miss Cole, and she rose up in her wrath and beat a hasty retreat.

One evening Maria got the idea into her head that she would never see No. 4, Prince's Row again. She imagined herself much worse, and she was afraid to die because she reasoned that the worst pain she had suffered with her face would be nothing to what was in store for her. "I'm pegged out," she groaned aloud in her anguish of mind. "I wish I 'adn't bin so set on bein' a hinfidel. Uncle Peter 'ad to work 'ard to keep a Christian, but I reckon e's got the best now, after all."

When Miss Cole called in the afternoon she found Maria in a state to be converted.

"You might make a Christian of me," she said, with an hysterical sob. "You see, I've been thinkin' if I die a hinfidel I shan't 'ave a chance of seein' Uncle Peter again, an' poor ole man, e'd be so disappointed."

### Suggestions for Private Nurses.

### NIGHT WORK.

Remember that the night is for sleep; therefore any essential interruptions such as food-giving, medicine, temperature, bed-pan giving, must all be done as restfully as possible. Try to keep the state of repose as late as possible, eight or nine a.m. quite. It does not at all follow that because the remainder of the household is up and doing, or because the night-nurse has to go to her breakfast that the patient need be roused. Let the nurse coming to relieve her enter noiselessly, and without a word change places, leaving the shaded candle or candle-lamp still burning and the curtains drawn. It seems incredible, but I have heard of young hospital nurses, fresh from their training, keeping to hospital hours for their private patients, and beginning to wash and prepare them at six and seven o'clock! With the doctor coming at ten o'clock, nine o'clock will be soon enough, or even later when his visits are to be eleven or past. It is a great help to a weak patient not to begin the day too soon.

Do not talk to your patients during the night beyond what is absolutely necessary, the effort of bringing their attention to hear what you say (especially in the case of old people, rather deaf) may drive away the sleepy feeling.

Take care that the light is shaded from the ceiling, so that the patient on waking sees no brightness, and if the nurse keeps out of sight, not moving about if she suspects the patient is waking, then the eyes are far more likely to gently close again and sleep return once more, whereas there might be talking if the nurse were seen. Some patients like to know by nurse's shadow on the wall that at least she is there, because it makes them feel safe and restful; if so, humour them. It is no true kindness to come forward officiously each time you think the patient is awake with inquiries as to how he feels, or if he wants a drink. By morning he might tell his friends that "nurse was most kind and attentive," but I think she would have proved herself a still better nurse if she had effaced herself more.

But when the nurse is desirous the patient should wake for food or medicine, then is the time to be moving about, seeing to the fire, adding to the steam kettle, getting the food ready, in order that the patient may gradually rouse instead of having to be spoken to or touched. It is much better to do these necessary little things first, than to have to be seeing to them when the patient ought to be dropping off to sleep once more, and the nurse ought to be perfectly quiet. In fact, no nurse should think it a grievance if once in a way it is necessary to put the light out so that the patient may doze off again. Often a quarter of an hour is quite sufficient, and then the nurse can quietly rekindle her light. Much more might be said about attending to the fire quietly, and other details, but common-sense will help in these matters if every nurse remembers, as a golden rule of paramount importance, that "Night is meant for rest."

### Appointments.

Taunton and Somerset Hospital.—On August 9th Miss E. Gertrude Evans was appointed Housekeeper. She was trained at Leeds General Infirmary, and has subsequently been sister at Monsall Hospital, Manchester; sister at Royal Chest Hospital, City Road; and charge nurse at the Western Hospital, Fulham.

Ryde County Hospital.—On August 9th Miss Carr was appointed Sister of Children's Ward. She was trained at the Royal Hants County Hospital, Winchester, and has since been acting as charge nurse in Winchester Hospital.

Spittlesea Infectious Hospital, Luton.—Miss E. Annie Whitcombe has been appointed Matron. She was trained at Westminster Hospital, and has since been on the private nursing staff.

Indian Army Nursing Service.—Miss Ada M. Harris has been appointed nursing sister. She was trained at the Royal Free Hospital, London, and since November, 1897, has been nursing plague cases in Bombay.

### Everybody's Opinion.

[Correspondence on all subjects is invited, but we cannot in any way be responsible for the opinions expressed by our correspondents. No communication can be entertained if the name and address of the correspondent is not given, as a guarantee of good faith but not necessarily for publication, or unless one side of the paper only is written on.]

### A NURSE AT EARL'S COURT EXHIBITION.

"CURIOSITY" writes: I see an announcement in the *Standard* that "Nurse Robinson will attend every day this week from two till ten p.m. at Byards, E. Citrivdora Stall in the Queensland Court of the Earl's Court Exhibition." Do you know what it means? Is the nurse on show, or is she in attendance in case the great wheel should break down, or some other catastrophe occur?

### NURSES IN PRIVATE CASES.

"A PRIVATE PATIENT" writes: I wonder whether it is the general experience that nurses in private cases are not as well treated as they ought to be. I have been in the care of four nurses at different times, and when I have told them how highly I think of nurses in general and how very much we invalids owe to them, the reply has always been, "I only wish other people thought as you do, for very often we get very scant consideration and have to put up with a good deal of discomfort."

### NURSES' DRESS.

"VERA" writes: As an old nurse my soul is often grieved by the startling and quaint costumes which I meet in the street, worn by persons apparently purporting to be nurses. I constantly see nurses dressed in what I can only characterise as "fly away" garments, tiny, ridiculous bonnets, fanciful dresses, a great expanse of apron, dangling chatelaines, and what not, and very often chains and bracelets also! Just as there is a "fitness in things" as far as regards nurses' conversation, so also is there a fitness to be observed in their clothes to my mind. Perfect neatness, absolute unobtrusiveness, and quietness seem the only right rules for a nurse's dress. To be inconspicuous is infinitely better than to flaunt around in enormous veils, enormous fringes, long trains, and inappropriate garments. Cannot nurses who love and honour their profession save it from the opprobrium which fanciful and unsuitable dressing brings upon it? I am sure it is often want of thought that leads nurses into absurdities of attire, and that they would be the first to see the inappropriateness of these absurdities if it were only pointed out to them.

### DO NURSES SMOKE?

M. G. S. writes: In answer to a paragraph in "The Open Letter" a few weeks ago, I write to say that some nurses do smoke, and I think the habit is not peculiar to the minority. Is it a very shocking or derogatory confession? "Mannish" is surely a term that is quite obsolete in connection with the subject. The niceness of our personalities is so important an item in our private training that the idea of any of us being supposed to "reek of stale smoke" is simply absurd. To efface the trace of a decent tobacco is a small affair—only a matter of the toothbrush and water. No one disputes the disinfecting properties of the weed but those who are ignorant of its qualifications. Bar us from the use that we may enjoy sore throats in preference. Nurses have hundreds of irritating, trifling worries that wear like the dropping of continual water. Our training does much to fortify the character, but when nerves are overstrung and sleep is broken, the effect of a cigarette is of immediate and soothing relief. Our pleasures are not so numerous that we should be debarred from one which, pursued with moderation, at the right time, and in the proper place, gives our patients no annoyance, and is not only harmless to ourselves, but enables us to return to our invalid bright and cheerful. Nothing is of more immediate benefit to the sick than to be tended by those possessed with a sweet content and a happy serenity of manner; and it is my opinion that a cigarette is of great assistance to preserve this demeanour so essential.

### THE HACKNEY MATERNITY HOSPITAL AND NURSING INSTITUTION.

IN reference to the fire at this institution the WARDEN writes that the report in the daily papers was not correct. She says: The fire originated in a nurse's private bedroom, where one little baby ten months old adopted by the nurse when the mother died was sleeping. No one else was in danger and no one was hurt except that one nurse burnt the tips of her fingers. The fire was extinguished before the arrival of the fire engine through the efforts of Police-constable Gould 411 and some Salvation Army bandsmen who were outside holding an open-air meeting. All the same, it might have been serious, so I had the patients and babies carried into the isolation ward away from the house.

### BREAKFAST IN THE BEDROOM.

"AN OLD NURSE" writes: If your correspondent, "One Who Lives to Learn," intends to continue in private work, and wishes to be happy and make those around her so, she cannot too soon learn that she will have to take things as she finds them, and make the best of them. At one case she may find herself in most luxuriant quarters, where she will have liveried servants to wait upon her and bring her food in silver dishes. Her next case may be one where she will have no place at all to call her own, but will have to sleep, wash, dress, and take her food in her patient's room. These are two extremes which most nurses who have been any length of time in private work must have experienced. Of course, there are many nice cases between, and most ladies are anxious to do all they can to make a nurse comfortable. At the same time they expect her to accommodate herself to the ways of the house, and not give more trouble than is absolutely necessary.

"EDITH," answering the question, Is it usual to give nurses their breakfast in the bedroom? writes: My experience is that it is generally so. If a nurse refuses to take her meals in the kitchen they are served to her in her own or her patient's room. Although she may be, both by birth and education, their superior, very few of those who employ her will sit down to the table with a nurse on terms of equality. I am a private nurse of fifteen years' standing, and was brought up and educated in a refined home; yet when nursing I have frequently had nowhere to bath or dress, except my patient's room, where also I have been expected to eat and sleep, and that not because it was an infectious or serious illness, but simply that I was not thought to require any other room. A private nurse is expected to have no likes or dislikes, never to be tired or cross or sleepy; to always be willing and ready to meet any demands made on her time and patience; to eat whatever is given her—anywhere and anyhow. I worked for one of the largest and oldest nursing institutions in London for several years, and that was my experience. For some years now I have taken cases for myself, and would on no account again work from an institution. I make my own terms, and go nowhere where I am not received as one of the family, and kindly treated. There is a world of difference between working for an institution and for yourself. I do not wear any uniform, but dress as any other lady would. I ask and obtain a high salary—what was called in THE HOSPITAL a short time since as one of the "plums of the profession." But the life is not what it is painted by outsiders, and no one should enter the field of private nursing who does not love her work. It is a self-denying life—a giving up of all your own wishes, always doing as others please. The last in the house to be thought of when there is any pleasure to be given, the first when there is trouble of mind or body, sorrow or sickness, or sympathy and help is required. So the life has its compensations.

### To Nurses.

IN order to increase and vary the interest in the *Mirror*, we invite contributions from any of our readers in the form of either an article, a paragraph, or information, and will pay a minimum of 5s. for each contribution. All rejected manuscripts are returned in due course, and all payments for manuscripts used are made at the beginning of each quarter, *i.e.*, January 1st, April 1st, July 1st, and October 1st.

### For Reading to the Sick.

"OUR HEAVENLY HOME."

**Verses.**

Thus Heaven is gathering, one by one, in its capacious breast,
All that is pure and permanent, and beautiful and blest;
The family is scattered yet, though of one home and heart,
Part militant in earthly gloom, in heavenly glory part.
But who can speak the rapture when the circle is complete,
And all the children sundered now before their Father meet?
One fold, one shepherd, one employ, one everlasting home:
"Lo, I come quickly!" Even so, amen, Lord Jesus come.
—*E. Bickersteth.*

The sands of time are sinking,
The dawn of Heaven breaks,
The summer morn I've sighed for,
The fair sweet morn awakes;
Dark, dark hath been the midnight,
But day spring is at hand,
And glory, glory dwelleth
In Immanuel's land.

The King there in His beauty
Without a veil is seen;
It were a well-spent journey,
Though seven deaths lay between;
The Lamb, with His fair army,
Doth on Mount Zion stand,
And glory, glory dwelleth
In Immanuel's land.

O, Christ He is the fountain,
The deep sweet well of love!
The streams on earth I've tasted,
More deep I'll drink above;
There, to an ocean fulness,
His mercy doth expand,
And glory, glory dwelleth
In Immanuel's land. —*Cousins.*

Oh, then what raptured greetings
On Canaan's happy shore,
What knitting severed friendships up
Where partings are no more!
Then eyes with joy shall sparkle,
That brimmed with tears of late,
Orphans no longer Fatherless,
Nor widows desolate.

Bring near Thy great Salvation,
Thou Lamb for sinners slain,
Fill up the roll of Thine elect,
Then take Thy power and reign;
Appear, Desire of Nations,
Thine exiles long for Home,
Show in the heavens Thy promised sign,
Thou Prince and Saviour come. —*Alford.*

**Reading.**

In that Home we shall find leisure to enjoy companionship—the companionship above all of Christ Jesus, our Lord and King; the companionship secondly of angels and saints and long-loved earthly friends. There will be no hurrying pressure of work and engagements in heaven; no intercourse cut short for want of time. In the grand reposeful leisure of eternity friendships will develop, minds will gather rich stores from other minds, and heaven will be a place of abundant occupation as well as of abundant leisure. What the manner of the "service" shall be we cannot yet know. It will be in its every part a distinct carrying out of the will of God, with no hesitation, no reluctance, no distaste. The very essence of our life there will be a spirit of joyous obedience.—*Agnes Giberne.*

### Notes and Queries.

**The contents of the Editor's Letter-box have now reached such unwieldy proportions that it has become necessary to establish a hard and fast rule regarding Answers to Correspondents. In future, all questions requiring replies will continue to be answered in this column without any fee. If an answer is required by letter, a fee of half-a-crown must be enclosed with the note containing the enquiry. We are always pleased to help our numerous correspondents to the fullest extent, and we can trust them to sympathise in the overwhelming amount of writing which makes the new rules a necessity.**

**Every communication must be accompanied by the writer's name and address, otherwise it will receive no attention.**

*Boys' School.*

(174) Will you kindly answer the following? I have had seven years experience of nursing and three years' training, but as I have done private nursing since and then taken a long rest after influenza I cannot expect to obtain any hospital appointment. Will you tell me how I can get the post of nurse-matron at one of the large boys' schools? (2) also if there are any other institutes that supply nurses for the winter on the Riviera besides the Hollond Institute; also what would be the prospects of a nurse working on her own account at the Cape?—*Wee Mike.*

The *Guardian* and *Church Times* have a good circulation amongst schoolmasters, partly on account of their advertisements. It might be worth while to insert an advertisement in one or other of these papers at least three times. August is a good month to do so, as preparations must then be made for reopening schools after the long vacation. 2. The Elgin Institute has recently opened a branch from November to May at Alassio. The address is Miss Ellison, Châlet Santa Cruxe, Alassio, N. Italy. 3. The prospects are fair. Do not go unless you have friends there or have work already promised. For the latest particulars, however, you should apply to the Emigrants' Information Office, 31, Broadway, Westminster.

*Young Nurse.*

(175) Could you give me any advice under the following circumstances? I am just turned 21 years of age, and have had two or three cases of nursing, and could be well recommended by a clergyman and doctors, as well as by sisters of mercy. I should like to take charge of an invalid or to go into a hospital where a salary is paid. I am now in charge of a lady who is slightly demented.

Your age would prevent your being accepted by most of the larger general hospitals for training. Asylum work, however, is well paid, and you would probably have little difficulty in obtaining employment in an asylum either for imbeciles or lunatics. The Matron, the Schools for Imbecile Children, Darenth, Dartford, Kent, would inform you if she has a vacancy on her staff; and also the payment offered. When you are about twenty-four you could take your three years' training in general nursing. This course would cost nothing in money, and would open a very fair field as regards remuneration later.

*County Hospital.*

(176) Can you oblige a reader of The Hospital by giving the addresses of some county hospitals near London, not more than 30 miles away, where a lady probationer would be taken for three or four months to gain further insight into surgical and clinical work, by weekly payment?

Our correspondent encloses a stamped envelope for a private reply, without the 2s. 6d. fee. We have therefore to reply to her query in our open column for which no charge is made. Many hospitals give a short training to paying probationers, but none of them care for this class of work; it is unsatisfactory both to the institution and to the probationer. For a list of county hospitals, and the conditions of training offered by them, we refer her to "The Nursing Profession: How and Where to Train," price 2s., from the Scientific Press, London.

*Preferment.*

(177) I would feel so much obliged if you would give me a little advice on the following: I am superintendent nurse at a Poor Law infirmary of 250 beds, and 150 for insane patients, making a total of 400 beds. As I am anxious to become a matron of a large hospital, I would like to know if I would be considered as eligible as a matron from a small hospital; or if preference would be given to the latter? Would you advise me to get an appointment as matron of a small hospital, or keep the one I have at present, to gain the end I have in view?—*Superintendent Nurse.*

Keep your present appointment by all means, and endeavour to obtain practical insight into matrons' work by assisting your present matron if possible.

*Nurses' Co-operation.*

(178) Could you kindly inform me if there are any co-operative associations of nurses in London, and also where they are situated?—*A Birmingham Nurse.*

The nurses' co-operation is at 8, New Cavendish Street, W. The Society of Chartered Nurses, 24, Princes Street, Cavendish Square, W., is also worked on the co-operative principle. The members must be registered members of the Royal British Nurses' Association, 17, Old Cavendish Street, W.

*Oxygen Home.*

(179) Will you kindly give me the address of the Oxygen Home? I have a patient that I think would benefit by treatment there, and I wrote ten days ago to ask for rules, &c., and addressed letter to Fitzroy Square, London, but have received no answer. Can you tell me if it is still carried on?—*Nurse Ed th.*

The Oxygen Home, Fitzroy Square, London, W., is still carried on. Always be cautious not to interfere with treatment. Advising as to treatment is beyond a nurse's province.

### ANSWERS REQUESTED.

*Broom Tea.*

(180) Will someone kindly tell me the best way to make broom tea? When I make it, it goes such a dark colour and so very thick that I always strain it.—*An Anxious Maiden.*

### Travel Notes.

By Sister Grace.

### XXXII.—GRINDELWALD.

Grindelwald may be easily visited from Interlaken, or a stay may be made there on fairly reasonable terms. It is a place of a totally different character to Interlaken, and its charms consist in its fine mountain scenery and the opportunities for walking and climbing it affords to the energetic. Amusements there are none, and shops are few and expensive. If you are not climbers, or at least very good walkers, you may see all that is available for you in a day's excursion from Interlaken.

### To Visit Grindelwald from Interlaken.

You take the train direct; return fare, 4 francs 80 cents. A carriage to Grindelwald and back, with two horses, only costs 25 francs, so that if you are a party of five the expense is exactly the same and the pleasure greater. It is by no means a hard or fatiguing walk, and quite within the powers of moderate pedestrians. I can do it with great ease, and I am by no means a good mountaineer. The road and the rail follow nearly the same route, and whatever way you go it is a delightful journey. You ascend the Black Lütschine and pass along the Lütschenthal under the slopes of the Schynige Platte, though not so near as by the road. After Lütschenthal the ascent is made by the rack and pinion method. Here you begin to see the Wetterhorn and the Berglistock, then the Eiger, the Schreckhorn, the Jungfrau, and the Silberhorn, with the Finsteraarhorn in the distance.

### Accommodation in Grindelwald.

If for the sake of the walking and the splendid air of the mountains you decide to make a stay, you may do so reasonably at the Hotel Pension Grindelwald, terms from 5 fr. per day, or at the Hotel Pension Schönegg, from 5 to 10 fr. per day.

### The Glaciers.

The one day tourist usually contents himself with visiting the upper glacier only, where the ice grotto is also to be seen. This latter, which is artificially hewn, gives me no pleasure at all, though I observed it was very popular with light-minded tourists. This glacier is quite near, reached easily in an hour. The lower glacier, the position of which has greatly shifted, is reached by the Bäregg; one sees thus the grand gorge of the Lütschine. A guide is unnecessary. One passes the church on the road to the glaciers, in itself an insignificant structure, and with the usual chilly Zwinglian interior, but it has a melancholy interest of its own from the number of those buried in the churchyard who have perished on the surrounding mountains.

### Mountain Excursions.

The ascent of the Faulhorn is not difficult if you are good walkers. The ascent will take five hours, and the descent three. A guide is necessary, unless you have with you one who knows the district well. Another expedition, a trifle shorter, but always requiring a guide, is from the Schynige Platte to the Faulhorn, starting from the Wilderswyl station, quite easily done in a day. One of the best walks is from Grindelwald to Meiringen over the great Scheidegg; it is twenty miles, but the walking is good. The distance may be greatly shortened by stopping at Rosenlaui for the night. The spot is most exquisite. Walk down to Meiringen in the early morning and return by boat and train. No guide is required if there are any good mountaineers in your party.

### Lauterbrunnen and Murren.

This is an easy excursion from Grindelwald, and it can even be done in a day from Interlaken. There is a junction of the Lauterbrunnen and Grindelwald lines at Zweilütschinen. The valley of Lauterbrunnen is about half a mile wide and is full of small waterfalls, which descend over the rocks in all directions. If you are staying at Grindelwald it is better to divide the expedition in two and see Mürren another day, though it is quite possible to see both in one. The first thing is to visit the Staubbach Fall. It has a drop of 980 feet and falls in a mighty curtain of fine spray. After this walk on to see the Trummelbach Fall; I prefer it to the Staubbach. There is a great deal to be seen in the Lauterbrunnen Valley which I have no space to tell you of, but must pass on to Mürren, which one reaches by cable and electric rail in an hour from Lauterbrunnen; return fare, 6 francs, or 3 francs 50 cents to ascend—you might like to walk back, probably. Once reached Mürren is an ideal spot in fine weather, but in rain everything is enveloped in a thick mist, and its melancholy is enough to haunt one for ever, and I think even Mark Tapley would be filled with sadness. On a clear, hot June day it is paradise, and though walks and expeditions are numerous, it seems enough to lie lazily on the slopes of the Allmendhubel and do nothing and think of nothing.

### Hints to Travellers.

It is a very unwise plan to leave changing your money when going abroad until you are on the steamer. You then receive the bare sum, without any reference to the rate of exchange. Get all this settled beforehand by Messrs. Cook or Gaze. In France the money seems to go farther than in England, because 25 francs go to a sovereign, and for practical purposes a franc equals a shilling. That is to say, if a thing is marked half-a-franc it is about what you would give 6d. for in England, and an article marked 10 francs would be 10s. in England. In Germany one does not gain in this way; a mark is exactly the same as a shilling. For a short tour take what money you think yourself likely to need and £5 over for an emergency. It is unnecessary to spend it, but it is very awkward and sometimes dangerous to be stranded in a foreign country without means. It is always advisable to have a passport; it costs a mere trifle—only 3s. 6d.—and it is useful in claiming letters at the Poste Restante. One word about the matter of letters. I have been told by foreign officials that many letters are lost by our English habit of putting "Esqre." on letters to a gentleman. It is regarded as a surname, and thus mistakes arise.

---

### TRAVEL NOTES AND QUERIES.

St. Jacut (Archer).—Yes, there is a convent, but it is extremely full in July, August, and September. The rules are not very stringent, but I think it would be unsuitable to you, as you have to be in your rooms at nine p.m.

Perugia (Sunflower).—Quite ideal for your purpose; the architectural sketches to be made there are innumerable. I think the book you want is J. Addington Symonds' "Sketches in Italy," in which he has an absorbingly interesting paper on Perugia. For hotels, there are the Grand Hotel Perugia, delightful, but expensive; and the Posta, more moderate. If you are remaining some time, and you say you can speak a little Italian, it would be worth while to take rooms. The walks outside the town are beautiful in all directions, and the atmospheric effects, especially at evening time, most beautiful.

Bonn (Violin).—Yes, it is a very good educational centre, but whether better than Heidelberg I can hardly say. I should think there is little difference between the two for girls' education. Expenses at Heidelberg may come a trifle less perhaps; on the other hand, Bonn is more open and airy for continued residence, though nothing like so picturesque; all the pensions and apartments are on the lower ground at Heidelberg, and it is warm and enervating in summer.

Boarding at Margate (Invalid).—I think you may meet with what you want at the Hereward Boarding House, Cliftonville, or at the Carlton, Sweyne Road, Margate.

Cologne and Mayence (Pair Oar).—You leave Liverpool Street at 8.30 p.m., reach Harwich at 9.55, and the Hook of Holland at 5.5 the next morning, go on at 5.33 a.m., and reach Cologne at 12.2 p.m. If your time is limited you will see all that is important in Cologne after luncheon, stay the night there, and go on by the 8 a.m. steamer the next morning for Mayence. The river is at its finest between Cologne and Mayence. Hotel at Cologne, Rheinischer Hof Unter Letterhennen, close to the cathedral; and at Mayence, Rheinischer Hof, good, but expensive; much cheaper, Hörns Pfalzer Hof, in the Munster-Platz. After seeing the cathedral, and spending one day in the town, I think you will not care for a longer residence, but settle at Konigswinter or Bacharach, or perhaps Coblentz.

